# Supplementary material for: Seminoma and Embryonal Carcinoma Footprints Identified by Analysis of Integrated Genome-Wide Epigenetic and Expression Profiles of Germ Cell Cancer Cell Lines
Source: PLoS One. 2014 Jun 2;9(6):e98330. doi: 10.1371/journal.pone.0098330 (PMC4041891; doi:10.1371/journal.pone.0098330)
Supplement: File S1 — ZIP file containing DMRforPairs output for significant regions. Please start from the html files. (ZIP) [file pone.0098330.s008.zip › figures/28549.pdf]

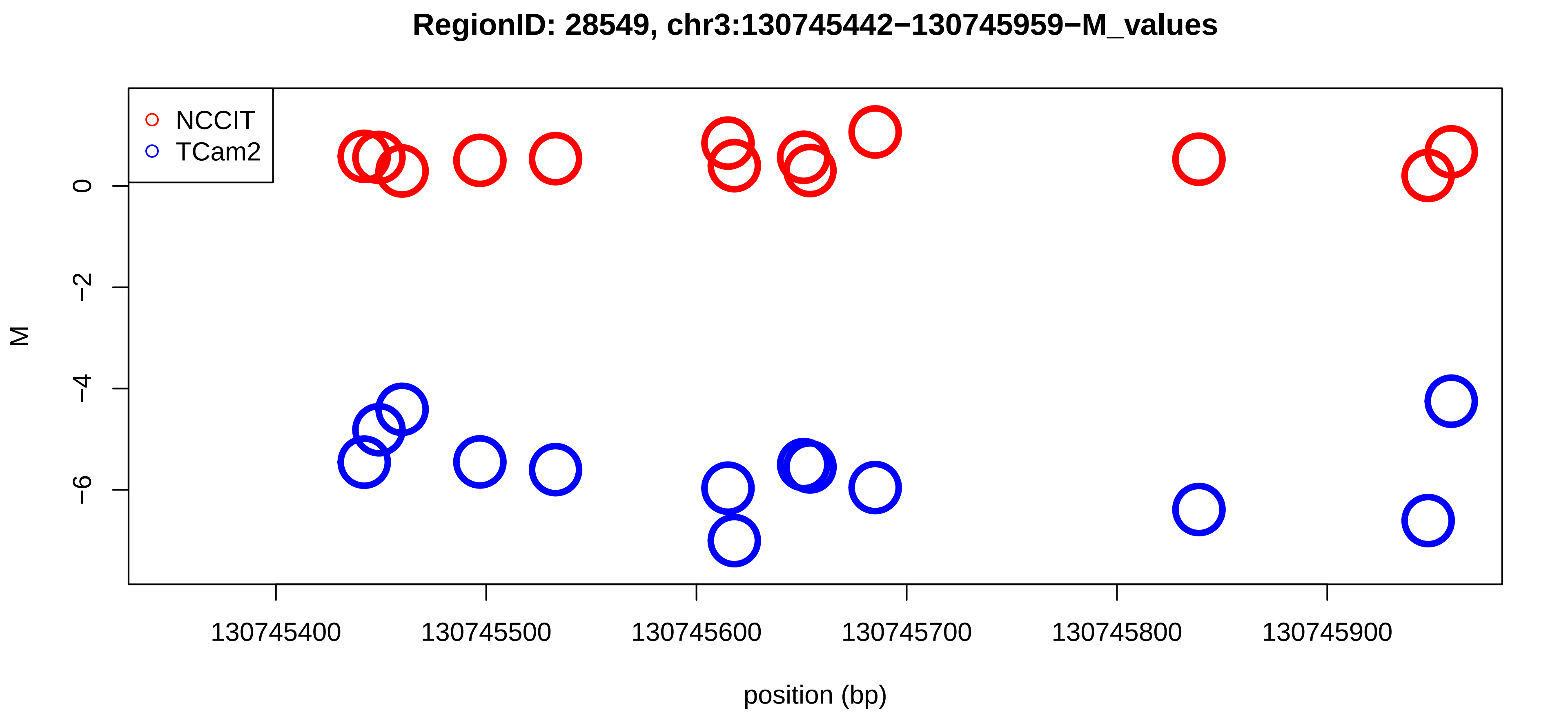

RegionID: 28549, chr3:130745442−130745959−M_values
NCCIT
TCam2
M
0
−2
−4
−6
130745400
130745500
130745600
130745700
130745800
130745900
position (bp)

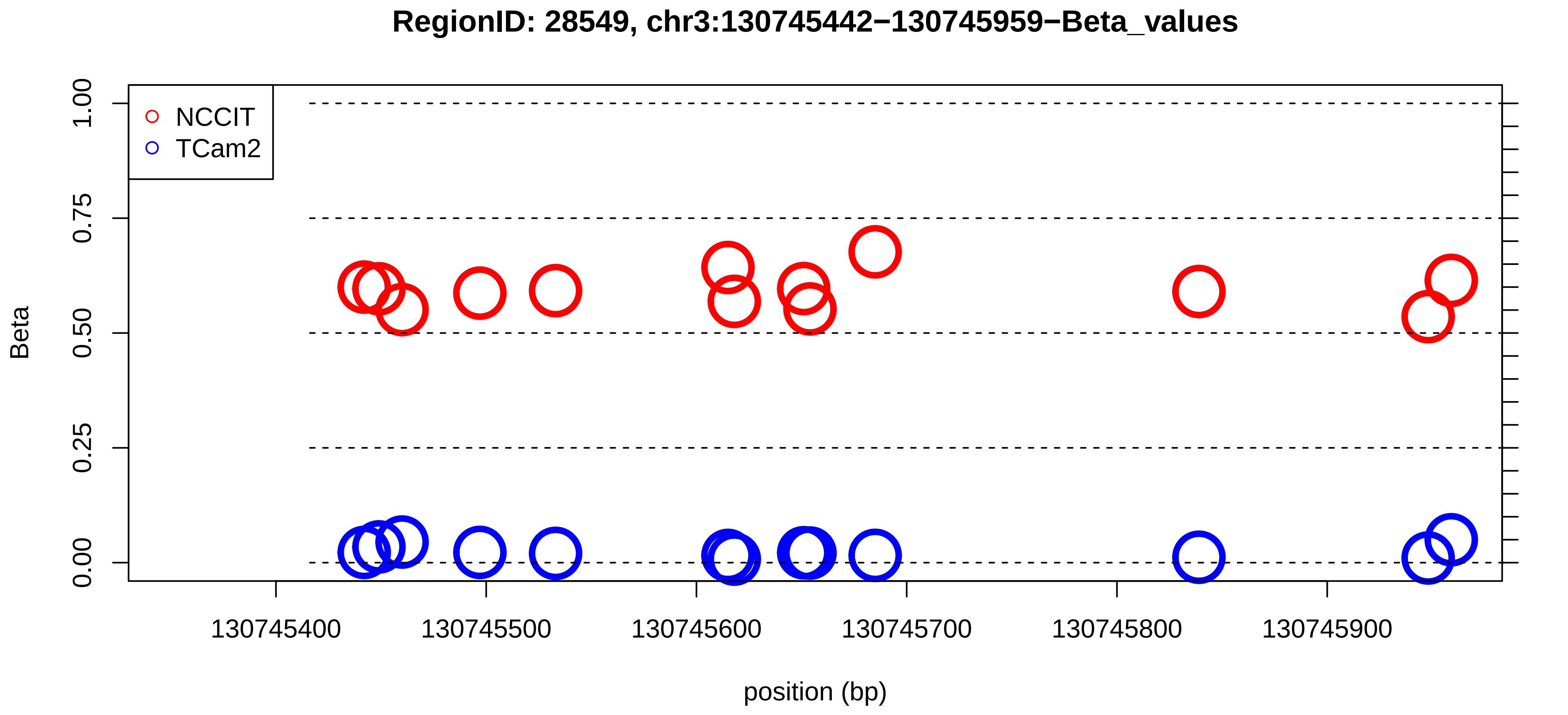

RegionID: 28549, chr3:130745442−130745959−Beta_values
NCCIT
TCam2
Beta
1.00
0.75
0.50
0.25
0.00
130745400
130745500
130745600
130745700
130745800
130745900
position (bp)

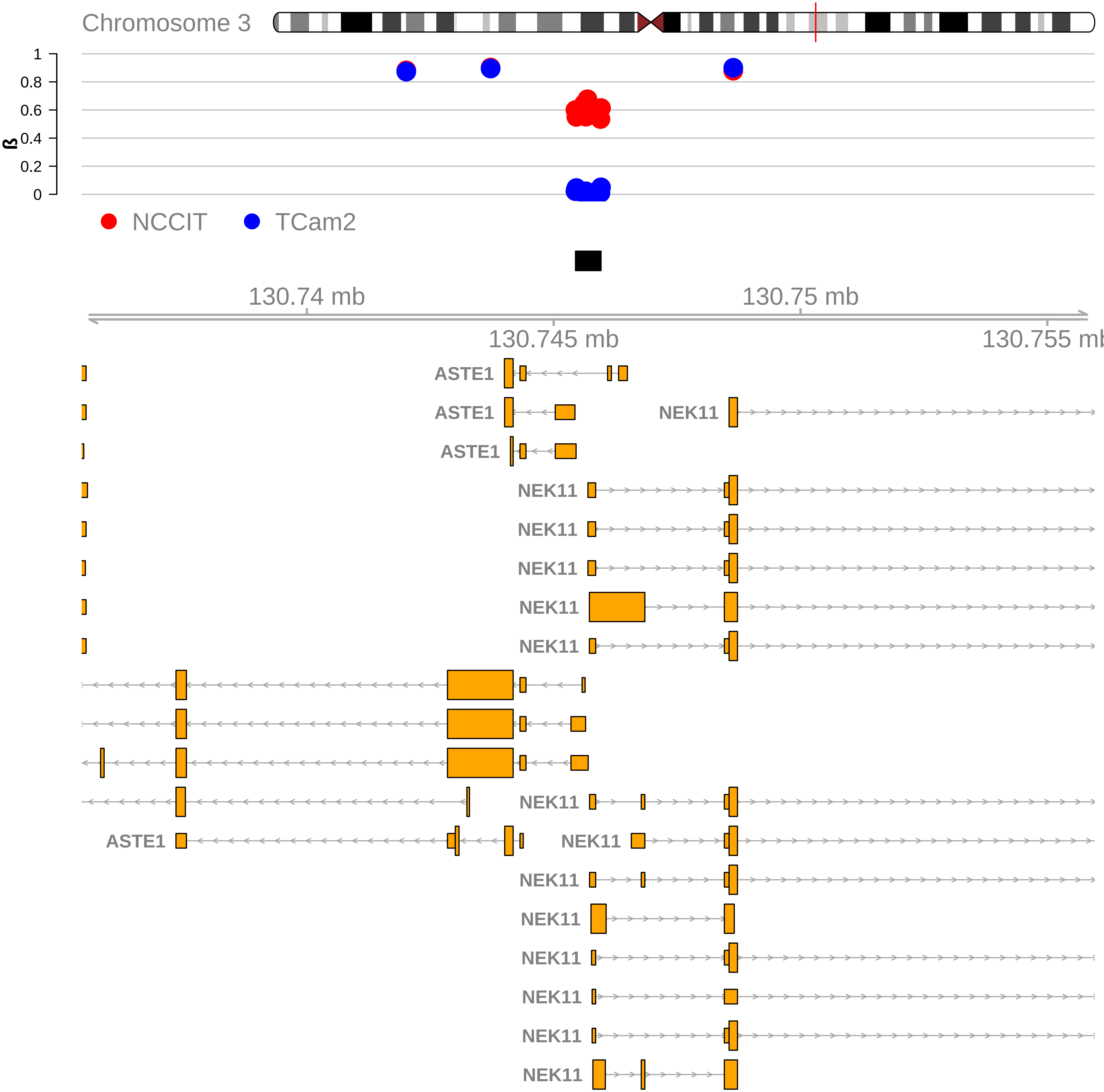

Chromosome 3
ß
1
0.8
0.6
0.4
0.2
0
NCCIT
TCam2
130.74 mb
130.75 mb
130.745 mb
130.755 mb
ASTE1
ASTE1
NEK11
ASTE1
NEK11
NEK11
NEK11
NEK11
NEK11
NEK11
ASTE1
NEK11
NEK11
NEK11
NEK11
NEK11
NEK11
NEK11
